# Precision genetic cellular models identify therapies protective against ER stress

**DOI:** 10.1038/s41419-021-04045-4

**Published:** 2021-08-05

**Authors:** Irina V. Lebedeva, Michelle V. Wagner, Sunil Sahdeo, Yi-Fan Lu, Anuli Anyanwu-Ofili, Matthew B. Harms, Jehangir S. Wadia, Gunaretnam Rajagopal, Michael J. Boland, David B. Goldstein

**Affiliations:** 1 Institute for Genomic Medicine, Columbia University Irving Medical Center, New York, NY USA; 2 Janssen Prevention Center, Janssen Pharmaceutical Companies of Johnson & Johnson, San Diego, CA USA; 3 Janssen R&D US, San Diego, CA USA; 4 Discovery Sciences, Janssen R&D, Spring House, PA USA; 5 Department of Neurology, Columbia University Irving Medical Center, New York, NY USA; 6 Department of Genetics and Development, Columbia University Irving Medical Center, New York, NY USA

**Keywords:** Stress signalling, Cellular imaging, High-throughput screening, Neurodevelopmental disorders

## Abstract

Rare monogenic disorders often share molecular etiologies involved in the pathogenesis of common diseases. Congenital disorders of glycosylation (CDG) and deglycosylation (CDDG) are rare pediatric disorders with symptoms that range from mild to life threatening. A biological mechanism shared among CDG and CDDG as well as more common neurodegenerative diseases such as Alzheimer’s disease and amyotrophic lateral sclerosis, is endoplasmic reticulum (ER) stress. We developed isogenic human cellular models of two types of CDG and the only known CDDG to discover drugs that can alleviate ER stress. Systematic phenotyping confirmed ER stress and identified elevated autophagy among other phenotypes in each model. We screened 1049 compounds and scored their ability to correct aberrant morphology in each model using an agnostic cell-painting assay based on >300 cellular features. This primary screen identified multiple compounds able to correct morphological phenotypes. Independent validation shows they also correct cellular phenotypes and alleviate each of the ER stress markers identified in each model. Many of the active compounds are associated with microtubule dynamics, which points to new therapeutic opportunities for both rare and more common disorders presenting with ER stress, such as Alzheimer’s disease and amyotrophic lateral sclerosis.

## Introduction

The study of rare monogenic disorders has yielded a number of insights into the molecular mechanisms underlying the pathobiology of more common diseases [1, 2]. Numerous diseases affecting both the central and peripheral nervous system involve elevated endoplasmic reticulum (ER) stress [3, 4]. In particular, ER stress has been implicated in diseases including Parkinson’s disease, Alzheimer’s disease, and amyotrophic lateral sclerosis (ALS) [5]. This suggests that therapeutic agents that ameliorate the effects of ER stress in monogenic disorders could have benefits across a broad range of disorders. Screens to identify such agents in the context of complex neurodegenerative diseases are challenging to implement; however, the etiology of a number of monogenic diseases is in large part attributed to ER stress including the congenital disorders of glycosylation (CDG) and deglycosylation (CDDG) [6–8]. Of particular interest, mutations in *PMM2*, the gene that encodes phosphomannomutase 2, result in the most common CDG [9]. Studies suggest that in PMM2*-*CDG, cells with weaker ER stress responses are more vulnerable to damage than cells with stronger ER stress responses [10]. Moreover, mutations in *DPAGT1*, which encodes the target of the well-known ER stress inducer tunicamycin [11], result in another CDG with systemic phenotypes [12, 13].

Here we utilize a morphological profiling and screening paradigm to identify agents that protect against the cellular stresses resulting from CDG and CDDG causal mutations. We focus specifically on mutations in *PMM2* and *DPAGT1*, and in *NGLY1*, which causes the only reported CDDG [8]. We used genetic engineering to generate CDG and CDDG genotypes in a karyotypically normal human cell line in order to create cellular models amenable to mutation-specific phenotype identification. These CDG and CDDG cell lines were used in high-content small molecule screens to identify compounds that revert the imaging phenotypes caused by these mutations. Specifically, we screened 1049 annotated compounds representing a broad chemical space and multiple target classes. In order to validate the performance of the screen, we selected 16 compounds that were ranked amongst the best at phenotype reversion in the screen (protective compounds) and 10 compounds that did not affect aberrant phenotypes (non-active negative control compounds). We then evaluated these compounds in assays designed to test how well they revert mutational phenotypes in the three cellular models.

## Materials and methods

### CRISPR/Cas9 genome editing of hTERT RPE-1 cells

CDG and CDDG lines were generated by CRISPR/Cas9 genome editing of hTERT RPE­1 (ATCC, CRL­ 4000TM) at the Columbia Stem Cell Core Facility. Promoter (U6) and gRNA scaffolds were synthesized by IDT and cloned into the pCR-Blunt II-TOPO plasmid (ThermoFisher Scientific, cat. K280002). Nucleofector (Lonza) was employed to introduce gRNA and Cas9-GFP plasmids into hTERT RPE-1 cells. After nucleofection, single colonies were manually picked into either 96-well plates or 10 cm dishes, incubated for ten days to reach confluency (96-well plate) or visible colonies (10 cm dish). For each colony, DNA was extracted by the KAPA Mouse Genotyping Kit (KAPA Biosystems) and genotyped by Sanger sequencing.

Guide RNA scaffold and termination signal: GTTTTAGAGCTAGAAATAGCAAGTTAAAATAAGGCTAGTCCGTTATCAACTTGAAAAAGTGGCACCGAGTCGGTGCTTTTTTT.

gRNA *NGLY1:* GGTGATTGCCAGAAGAACTAAGG, *PMM2:* GAATTCAATGAAAGTACCCCTGG*, DPAGT1:* CATGATCTTCCTGGGCTTTGCGG.

### Chemicals

Tunicamycin (cat. 3516) and salubrinal (cat. 2347) were from Tocris. Rapamycin (cat. HY-10219) was from MedChemExpress. All screened compounds were provided by Janssen Pharmaceuticals.

### Proliferation measurements

Cells were seeded in 96-well tissue culture plates and treated the next day as described in “Results”. At the indicated time points, the MTT assay was performed as described [14]. One-way ANOVA multiple comparisons and Dunnett test (GraphPad Prism software, v.8.2.0) were used to determine the equality of the means of different samples. The confidence level (*p*) was 0.05.

### Quantitative RT-PCR

Total RNA was extracted by RNeasy Plus Mini kit (QIAGEN, cat. 74136) and reverse-transcribed with random primers using Superscript IV Reverse Transcriptase kit (ThermoFisher Scientific, cat. 18091200). One µL of cDNA was used in each qPCR reaction on a QuantStudio 5 (ThermoFisher Scientific) using SYBR Green PCR Master Mix (ThermoFisher Scientific, cat. 4364344). PCR primers detecting spliced and unspliced *XBP1* expression were as described [15], and for human *GAPDH* were ACAGTCAGCCGCATCTTCTT and TTGATTTTGGAGGGATCTCG. The relative expression levels of target genes were normalized to that of the reference *GAPDH* gene by using the ΔΔCt method [16]. The fold change in expression for each sample is relative to parental hTERT RPE-1 cells treated with vehicle.

### Immunoblot analysis

Cell lysates were prepared as described in [14], resolved in SDS-PAGE, transferred to PVDF membrane (Immobilon-P, Millipore, cat. IPVH00010), stained with appropriate antibodies (Supplementary Table 1) and developed as described [14]. Western blots were quantitatively analyzed via laser-scanning densitometry using NIH ImageJ v1.52k software.

### Immunocytochemistry

hTERT RPE-1 and the isogenic mutant lines were seeded on glass coverslips, fixed with 4% paraformaldehyde (PFA), permeabilized with 1% Triton X-100/PBS, blocked and stained in 1% BSA/ 0.1% Triton X-100/PBS and mounted in Prolong Antifade DAPI (Invitrogen). Antibodies and dilutions are listed in Supplementary Table 1. Imaging was performed on an inverted Zeiss AxioObserver Z1 fluorescent microscope equipped with an AxioCam 503 mono camera and filters for 405, 488, and 568 nm. Images were acquired with Zen 2 software and post-processing was performed with AdobePhotoshop CC.

### Senescence detection

Cells were seeded in 6 well plates, and stained using Senescence β-Galactosidase Staining Kit (Cell Signaling Technology, cat. 9860) according to manufacturer’s instruction. The images were acquired, and the number of stained cells was counted using Zeiss Primovert inverted brightfield/phase contrast microscope equipped with AxioCam ERc5s camera.

### Apoptosis detection

Cells were stained using APC-labeled Annexin-V (BD Biosciences, cat. 550474) and propidium iodide (PI) according to the manufacturer’s instructions and analyzed immediately on FACSCelesta (BD Biosystems). Data were processed using FlowJo v. 10.5.3 and Prism8 v8.2.0 software.

### Autophagy detection

Cells were collected by trypsinization, fixed with 4% PFA/PBS for 15 min at RT. Fixed cells were permeabilized using Intracellular Staining Permeabilization Wash buffer (BioLegend, cat.421002) according to the manufacturer instructions, stained with appropriate primary and secondary antibodies (Supplementary Table 1) and analyzed on a FACSCelesta cytometer (BD Biosystems). The data were processed using FlowJo v. 10.5.3 and Prism8 v8.2.0 software.

### Cellular morphology assessment by immunostaining

Cells were seeded in 96-well plates, treated with tested compounds, vehicle (DMSO) or positive controls. After 24 h, cells were fixed with 4% PFA/PBS, blocked with 1% BSA/PBS and stained with phalloidin-568 (ThermoFisher Scientific, cat. A12380). After two washes with PBS, cells were stained with 300 nM DAPI (BD Pharmingen, cat. 564907). Imaging was performed with an inverted Zeiss AxioObserver Z1 epifluorescent microscope equipped with an AxioCam 503 mono camera, and images acquired with the Zen 2 software. Post-processing was performed AdobePhotoshop CC software.

### High-content imaging and compound screening

The hTERT RPE-1 cells and *NGLY1*^−*/−*^*, PMM2*^*F119L/*^^−^*, and DPAGT1*^*+/−*^ mutant lines were plated in 384 well plates at a density of 3000 cells per well. The next day compounds were added to the cells at a final concentration of 10 µM and incubated for 24 h. Cells were stained with MitoTracker Red (Molecular Probes, cat. M7512) mitochondrial stain for 30 min according to the manufacturer’s protocol, then media was removed, cells were fixed in 4% PFA/PBS, permeabilized with 0.1% NP-40, and blocked with 3% BSA/ PBS overnight. For staining, ConcanavalinA-488 (Molecular Probes, cat. C11252), phalloidin-547 (Molecular Probes, cat. A22283), and DAPI were added to the wells, then washed before imaging. Images were acquired on a Molecular Devices Image Express microscope at 4 fields per well. Feature extraction from images was done with Perkin-Elmer Columbus Image Analysis software, and feature analysis and hit determination was performed using TIBCO Spotfire analysis package.

### Secondary validation of selected compounds

Candidate and control compounds from the high-throughput screen were validated using two assays, MTT and RT-qPCR for s*XBP1* expression as described above. Dose–response curves on hTERT RPE-1 cells identified the lowest non-toxic concentration for each of the candidate compounds. Cells were treated for 24 h with Group I (5.0 nM), Group II (1.0 µM), or non-active control (10 µM) compounds. Post treatment, total RNA was extracted by RNeasy Plus Mini kit for RT-qPCR. Alternatively, cells were subjected to MTT assay at days 0, 1, 3, and 5 post-treatment.

### Statistical analyses

Results are expressed as mean ± SEM for a minimum of three independent experiments. Sample size and statistical tests are detailed in the figure legends. Statistical analysis was performed using one-way ANOVA followed by Dunnett multiple comparisons post-test to compare each condition to vehicle-treated controls. *P* values ≤ 0.05 were considered significant.

## Results

### Establishment of precision human cellular models of CDG and CDDG

**Fig. 1:**
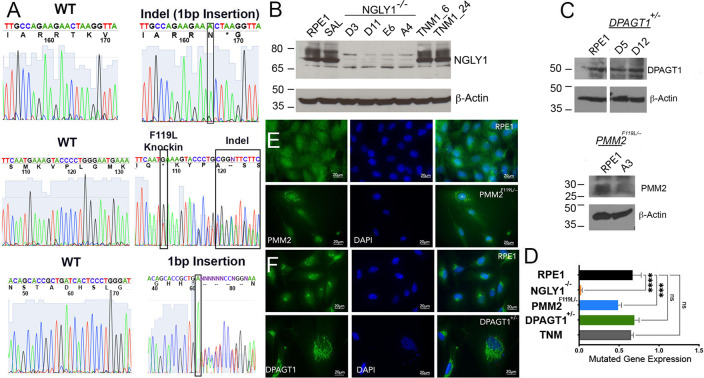
CDG and CDDG cellular models were validated by Sanger sequencing, immunoblot and immunofluorescence staining for target proteins. **A** Electropherogram traces for parental RPE-1 cells, CDDG—*NGLY1*^−/^^−^ D11 (top), and CDG—*PMM2*^F119L/^^−^A3 (middle) and *DPAGT1*^+/−^ D5 (bottom) lines. **B**, **C** Representative immunoblot images for target proteins in RPE-1 and isogenic CDDG (**B**) and CDG (**C**) lines. RPE-1 cells treated with TNM (1 µM for either 6 h or 24 h) were used as a positive control. **D** Quantification of the target protein levels in the edited RPE-1 cells (*n* ≥ 3 per genotype). Expression relative to levels in parental RPE-1 cells. ***P < 0.001, ****P < 0.0001, one-way ANOVA followed by Dunnett multiple comparisons post-test. **E**, **F** Representative immunofluorescence images for staining for PMM2 and DPAGT1 in parental RPE-1, CDG—*PMM2* ^F119L/^^−^ A3, and CDG *DPAGT1*^+/^^−^ D5 lines. Scale bar: 20 µm.

Genome editing was used to generate hTERT RPE-1 cell lines that mimic genotypes associated with CDG and CDDG. All known CDDG patients possess complete loss of function of NGLY1 [8]. We designed gRNAs to generate the recurrent *NGLY1* R401X missense variant, but after repeated attempts were unable to obtain the homozygous R401X genotype. Therefore, we screened clones for knock-out of *NGLY1* resulting from biallelic indel formation (*NGLY1*^−/−^). PMM2-CDG often results from compound heterozygous mutations that reduce enzymatic activity [6, 17, 18]. Compound heterozygous *PMM2* lines were generated by monoallelic knock-in of the second most recurrent and very severe mutation (F119L) [9, 19], and then screening for an indel on the second allele (*PMM2*^F119L/−^). We generated *DPAGT1*^+/−^ lines by monoallelic knockout via indel formation. All genotypes were confirmed by Sanger sequencing (Fig. 1A, Supplementary Fig. 1A–C, E).

As expected, *NGLY1*^−/−^ lines do not express NGLY1 protein and the levels of PMM2 in *PMM2*^F119L/−^ were decreased by ~50% (Fig. 1B–D). The expression level of DPAGT1 was different between the two *DPAGT1*^+/−^ clones analyzed despite confirmation of monoallelic disruption of *DPAGT1* (Fig. 1C and Supplementary Fig. 1C). Clone D5, however, consistently expressed ~50% of DPAGT1 relative to parental cells. This clone was used in the high-content screens discussed below.

Consistent with published studies [20, 21], we found DPAGT1 localized to the perinuclear space, and PMM2 was diffuse throughout the cytosol and nucleus (Fig. 1E, F, Supplementary Fig. 1D). Interestingly, PMM2^F119L^ was found in cytosolic puncta suggestive of protein aggregation (Fig. 1E). We failed to detect NGLY1 by immunocytochemistry using multiple NGLY1 antibodies (not shown).

### CDG and CDDG lines exhibit elevated ER stress and autophagy responses

**Fig. 2:**
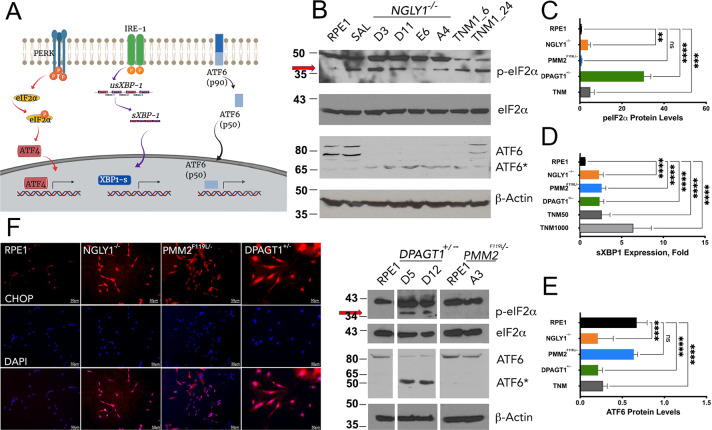
Cellular models of CDG and CDDG exhibit elevated ER stress responses. **A** Schematic of the three major ER stress pathways (analyzed markers are framed). **B** Representative immunoblot images for ER stress markers in RPE-1 and different clones of isogenic CDDG (top) and CDG (bottom) lines. RPE-1 cells treated with TNM (1 µM for either 6 h or 24 h) and salubrinal (SAL, 50 µM for 24 h) were used as negative and positive controls, respectively. **C** Quantification of p-eIF2α levels relative to total eIF2α protein (*n* ≥ 3 per each genotype or treatment). **D** Expression of spliced *XBP1* transcript (*n* ≥ 10 per each genotype or treatment). **E** Quantification of ATF6(90) levels in parental and edited RPE-1 cells. (*n* ≥ 3 per each genotype or treatment). Expression data in **C**–**E** are relative to levels in parental RPE-1 cells. *P < 0.05, **P < 0.01, ***P < 0.001, ****P < 0.0001, one-way ANOVA followed by Dunnett multiple comparisons post-test. **F** Nuclear localization of CHOP/ATF4 in parental and edited RPE-1 cells (scale bars are 50 µm).

Although a common molecular feature of CDG is elevated levels of ER stress [22], systematic examination of ER stress in CDDG has not been performed. In order to establish the ER stress profiles of the cellular models, we first established a baseline in isogenic RPE-1 cells using a moderate concentration of the N-linked glycosylation inhibitor and ER stress inducer tunicamycin [23, 24] and the ER stress inhibitor salubrinal [25]. We examined ER stress using markers from each of the three recognized pathways of ER stress (Fig. 2A): (1) detection of eIF2α Ser51 phosphorylation (p-eIF2α) and nuclear translocation of ATF4 and/or CHOP, (2) presence of spliced *XBP1* mRNA (s*XBP1*), and (3) cleavage of ATF6 [24, 26]. As expected, tunicamycin treatment resulted in strong induction of p-eIF2α, elevated expression of s*XBP1*, and reduced levels of ATF6(90) and corresponding increases of ATF6(60) due to cleavage (Fig. 2B–E, TNM).

All CDG and CDDG lines exhibited increased ER stress responses relative to untreated RPE-1 (Fig. 2B–F), but the distinct genotypes showed differential activation of the key ER stress response pathways. For example, *NGLY1*^−/−^ and *DPAGT1*^+/−^ lines exhibited activation of all three established ER stress pathways, whereas *PMM2*^F119L/−^ had only significant increases in *XBP1* splicing (Fig. 2B–E). In fact, the only pathway induced across all CDG and CDDG lines was splicing of *XBP1*.

**Fig. 3:**
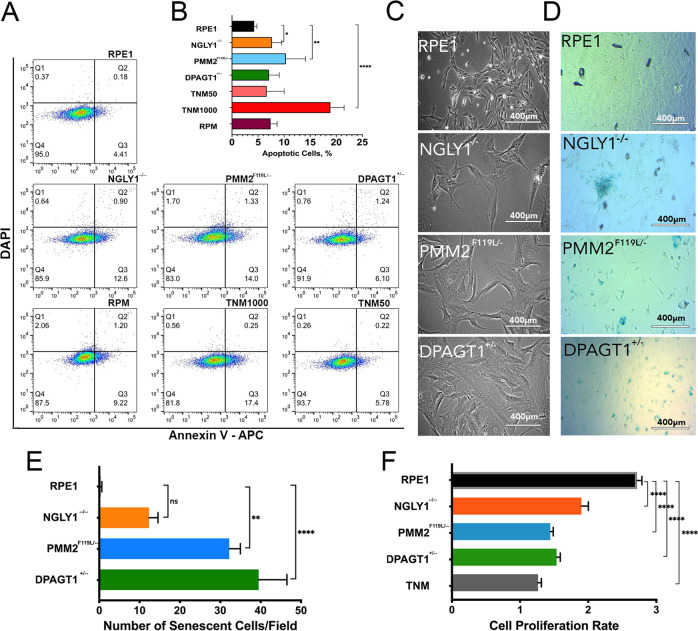
CDG and CDDG cellular models exhibit low levels of apoptosis, line-dependent levels of senescence and reduced proliferation. **A** Representative dot-blots of apoptosis detection (Annexin V staining) in RPE-1, CDG and CDDG lines. Tunicamycin was used as a positive control for apoptosis (1.0 µM, 24 h) and chronic ER stress (0.05 µM, 24 h). Rapamycin treatment (RPM, 500 nM) was used as a control for autophagy induction. **B** Quantification of apoptosis by flow cytometry (*N* = 3 experiments). **C** Representative phase-contrast images of cellular morphology. Scale bars, 400 µm. **D** Representative images of β-galactosidase senescence staining of parental RPE-1 and CDDG, *NGLY1*^−/−^ D11, and CDG *PMM2* ^F119L/−^ A3 and *DPAGT1*^+/−^ D5 lines. **E** Quantification of senescence levels as indicated by β-galactosidase staining (10 fields were counted per each genotype). **F** Quantification of cellular proliferation rates for CDG and CDDG lines relative to parental RPE-1 (*n* ≥ 10 per each genotype or treatment). To define cell proliferation rate, ratio of OD_590_ at 72 h to OD_590_ at 24 h post seeding was calculated. *P < 0.05, **P < 0,01, ****P < 0.0001, one-way ANOVA followed by Dunnett multiple comparisons post-test.

Low, but significant, levels of apoptosis were detected in *PMM2* ^F119L/−^ and *NGLY1*^−/^^−^ but not in *DPAGT1*^+/−^ lines (Fig. 3A, B). Apoptosis levels in mutant cell lines were comparable to low levels of induced ER stress (Fig. 3B, TNM50) and significantly lower than would be expected from high ER stress conditions (Fig. 3B, TNM1000) further suggesting that CDG and CDDG lines exhibit lower chronic ER stress responses.

**Fig. 4:**
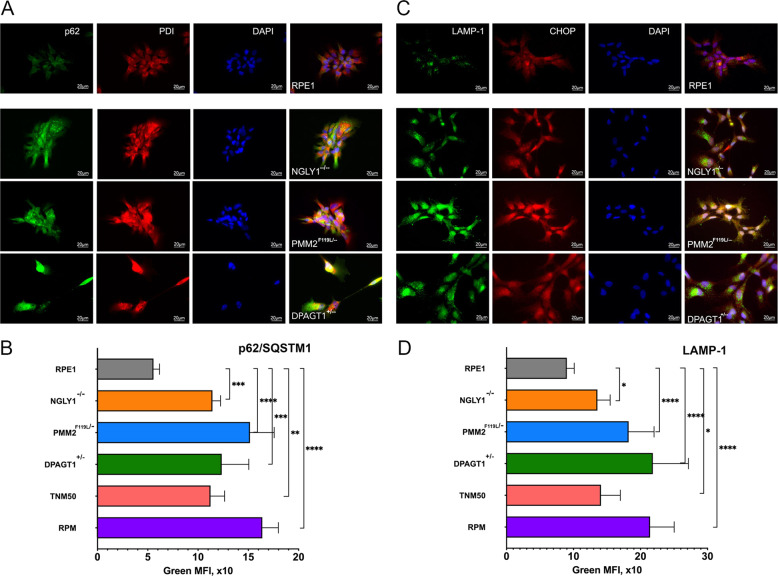
CDG and CDDG lines exhibit elevated autophagy levels. Representative immunofluorescence images of parental RPE-1 cells and CDDG and CDG cell lines stained with antibodies against **A** p62/SQSTM1 or **C** LAMP1 in combination with anti-CHOP or anti-PDI antibodies, respectively. Scale bar, 20 µm. Quantification of p62/SQSTM1 (**B**) and LAMP1 (**D**) staining by flow cytometry (*n* ≥ 3 per each genotype or treatment). *P < 0.05, **P < 0.01, ***P < 0.001, ****P < 0.0001, one-way ANOVA followed by Dunnett multiple comparisons post-test.

Autophagy is known to play an important role in the response to ER stress and is seen as a marker of chronic ER stress [27]. Significant upregulation of autophagy was detected in all mutant cell lines using markers of early (p62/SQSTM1) and late (LAMP1) stages of autophagy followed by fluorescent microscopy or flow cytometry (Fig. 4 and Supplementary Fig. 2).

### CDG and CDDG lines exhibit distinctive morphological phenotypes and proliferation defects

CDG and CDDG lines were characterized for phenotypes useful for high-content imaging screens. All mutant cell lines exhibited a flat, extended morphology (Fig. 3C) reminiscent of cellular senescence that was not seen in the isogenic parental line. Indeed, β-galactosidase staining confirmed various levels of senescence among the mutant cell lines (Fig. 3D, E). All lines demonstrated slower proliferation compared to the isogenic RPE-1 line (Fig. 3F). *DPAGT1*^+/−^ lines exhibited the slowest proliferation rates and were comparable to those observed in the parental line when subjected to chronic ER stress from low concentration tunicamycin exposure.

### Primary drug screen identifies compounds able to reverse CDG and CDDG cellular morphology phenotypes

**Fig. 5:**
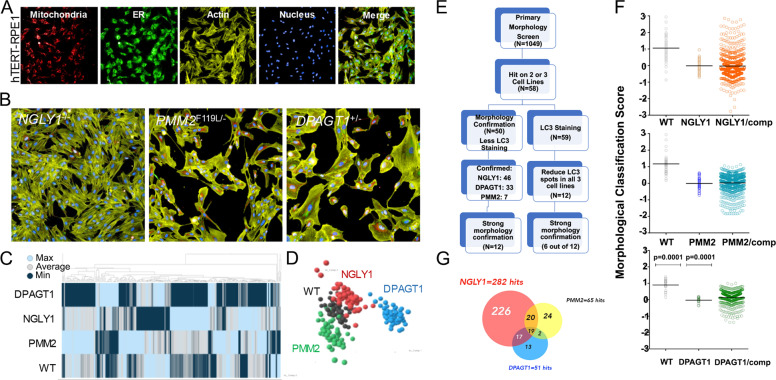
High-throughput drug screen against cellular morphology via cell painting identifies compounds able to revert aberrant line-specific morphological characteristics. **A** Cell painting images of parental RPE-1 cells. Cells were plated in 384-well tissue culture plate and stained with MitoTracker Red (mitochondrial stain), ConcanavalinA-488 (ER stain), phalloidin-547 (actin stain), and DAPI (nuclei) as described in the “Materials and methods” section. **B** Representative cell painting images of CDDG and CDG lines. **C**, **D** Hierarchical clustering and principal component analyses of extracted morphological features distinguishes CDG and CDDG cell lines from parental RPE-1 cells. **E** Screening workflow for selection of candidate compounds. **F** Representative scatter plots of primary screen results for each genotype. Each dot represents one well. For RPE-1 and vehicle treated isogenic CDG and CDDG cell lines, *N* = 56 replicate wells. For compound treatments, each compound was tested in *N* = 1 well. **G** Venn diagram comparing the number and overlap of compounds affecting each CDG/CDDG phenotype.

Our drug screening platform takes advantage of the distinctive cellular phenotypes that result from the CDG and CDDG mutations. In order to identify compounds able to correct aberrant morphological phenotypes in the mutant lines, we utilized a “cell painting” phenotypic assay [28, 29] based on stains for mitochondria, the actin cytoskeleton, endoplasmic reticulum, and nuclei (Fig. 5A). Machine learning algorithms were trained on acquired images of RPE-1 cells, and more than 300 cellular features, such as fluorescence intensity, presence and numbers of puncta, texture, and cellular shape and geometry were extracted and analyzed. Functional testing and validation of the cell painting assay was performed on CDG and CDDG cell lines (Fig. 5B). Importantly, hierarchical clustering and principal component analyses clearly distinguished mutant cells from each other and from parental RPE-1 cells (Fig. 5C, D). This demonstrates that there are distinct phenotypic, morphological changes that occur as a consequence of the CDG or CDDG mutation in each of the clones.

We screened 1049 annotated compounds representing a broad chemical space and multiple target classes on CDG and CDDG cell lines (Fig. 5E). The compound library was assembled with publicly available compounds that have known biological activities as well as Janssen proprietary compounds that have evidence of bioactivity compiled from multiple internal data sets. *NGLY1*^*−/−*^*, PMM2*^*F119L/*−^ *and DPAGT1*^*+/*−^ lines were treated for 24 h with 10 µM of each compound. Parental RPE-1 cells treated with vehicle (DMSO) served as a positive control while vehicle-treated CDG and CDDG lines served as negative controls (Fig. 5F). Post-treatment, the cell-painting assay was performed and a morphology score was computed for each compound’s ability to revert morphology of mutant cell lines toward that of parental cells. Results of the primary morphology screen identified 58 compounds that had positive effects in two or three cell lines (Fig. 5F, G). Because CDG/CDDG cell lines demonstrate elevated autophagy levels, primary screening hits were subsequently assessed for their ability to modulate autophagy by immunocytochemistry with LC3 as the marker. Twelve candidate compounds reduced autophagy in all three cell lines (Group I, Supplementary Table 2), and 10 additional candidate compounds (confirmed in at least 2 cell lines) that had a minimal, or no effect on autophagy (Group II, Supplementary Table 2).

### Evaluation of compounds for amelioration of ER stress and proliferation defects

The top six candidates from Group I and Group II were further evaluated their ability to alleviate ER stress in the CDG and CDDG lines. Compounds that improved phenotypes in all three genetic lines were also included for further testing. In order to validate the efficiency and potency of the primary screen, we selected 10 non-active compounds in the cell-painting assay for comparison to candidate compounds. In total, we tested 16 active compounds and 10 non-active control compounds for their effects on cell proliferation and s*XBP1* expression. We focused on s*XBP1* expression because it was the only ER stress marker dysregulated across all three models, and the only significant ER stress marker in *PMM2*^F119L/−^ (Fig. 2C–E).

**Fig. 6:**
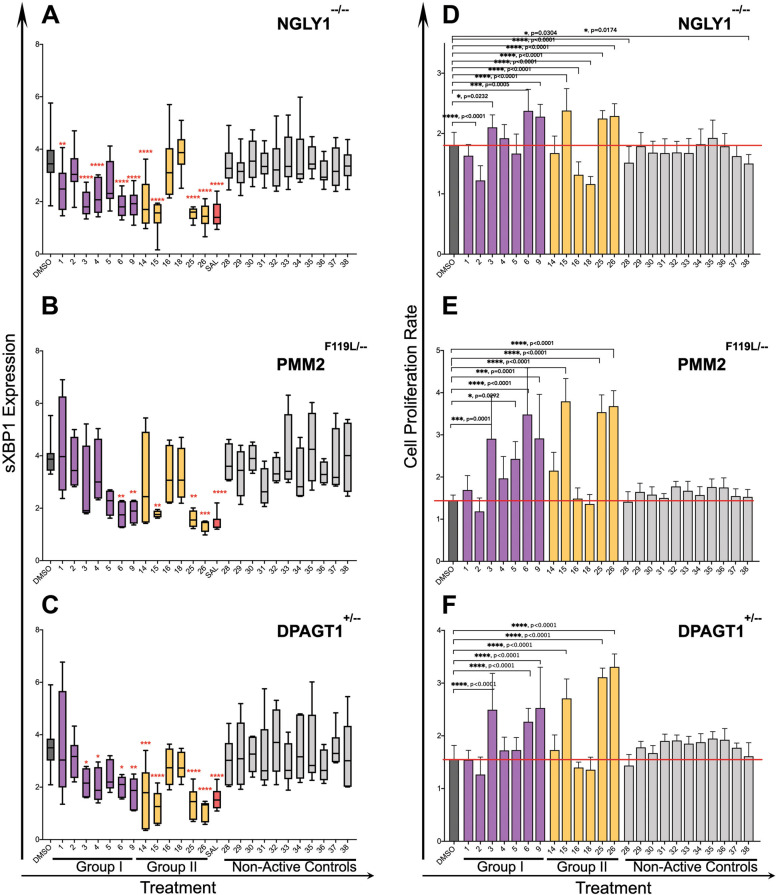
Validation that active compounds correct proliferation defects and an ER stress phenotype shared across the CDG and CDDG lines. CDG and CDDG cell lines were treated with optimized concentrations of candidate and non-active control compounds and expression of s*XBP1* and cell proliferation were assessed. **A**–**C** Expression levels of s*XBP1* in *NGLY1*^−*/−*^ (**A**), *PMM2*^*F119L/−*^ (**B**), and *DPAGT1*^*+/−*^ (**C**) clones treated with candidates or non-active compounds. Results are presented as a ratio of s*XBP1* levels in compound-treated cells to vehicle treated cells (*n* ≥ 10 per each genotype or treatment). **D**–**F** Modulation of proliferation rate of *NGLY1*^*−/−*^ (**D**), *PMM2*^*F119L/−*^ (**E**), and *DPAGT1*^*+/−*^ (**F**) lines treated with candidates or non-active compounds (*n* ≥ 10 per each genotype or treatment). To define cell proliferation rate, ratio of OD_590_ at 72 h to OD_590_ at 24 h post seeding (before treatment) was calculated. Data presented are an average for all clones available for a specific mutation. **P < 0.01, ***P < 0.001, ****P < 0.0001, one-way ANOVA followed by Dunnett multiple comparisons post-test.

All active compounds affected one or both assays in at least one of the cell lines (Fig. 6). Compound effects appeared to be mechanism and cell line-dependent in the proliferation and *sXBP1* assays. For example, Group I compounds showed effects on all lines (Fig. 6, purple bars) while the autophagy inducing compounds (Group II) were generally more efficient in the *NGLY1*^−/^^−^ and *DPAGT1*^+/−^ lines (Fig. 6, yellow bars). Compounds 1, 3, 4, 5, and 14 decreased s*XBP1* expression in CDDG lines compared to DMSO treated controls (dark gray bar) but did not affect proliferation (Fig. 6A, D). None of the ten non-active control compounds had an effect on s*XBP1* expression (Fig. 6A–C, gray) or proliferation (Fig. 6D–F, gray bars). It is interesting to note that salubrinal—a highly selective inhibitor of eIF2α phosphorylation—was as effective as some active compounds at reducing s*XBP1* expression. This is particularly interestingly for *PMM2*^F119L/−^ cells, which do not show induction of p-eIF2α.

We found no correlation between reduction of s*XBP1* expression and repair of proliferation for compounds 3, 4, 5, and 14 in CDG cell lines. Group I candidates 6 and 9 and Group II candidates 15, 25, and 26 all effectively reduced s*XBP1* expression (Fig. 6A–C), and showed restoration of proliferation in CDG and CDDG lines, (Fig. 6D–F). Active compounds, but not non-active controls, were able to revert aberrant cellular morphology similar to that of vehicle treated controls (Supplementary Fig. 3). Together, these data validate the effectiveness of the screen, and identify sets of compounds that are able to correct aberrant cellular phenotypes associated with CDG and CDDG genotypes.

**Fig. 7:**
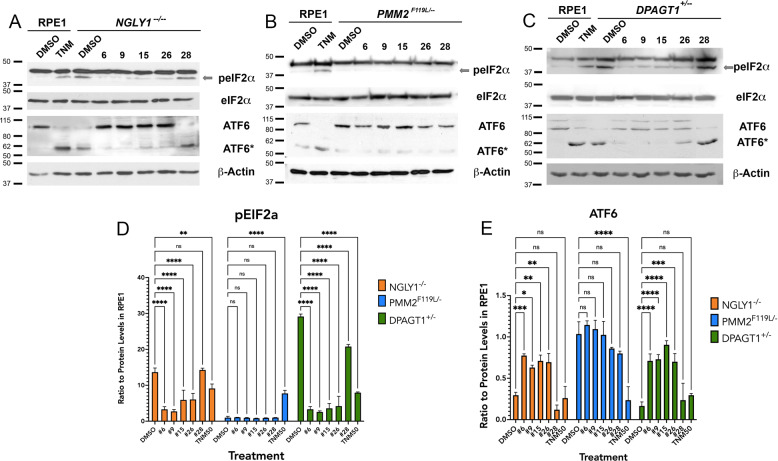
Select candidate compounds are able to alleviate the multiple ER stress phenotypes identified in each of the CDG and CDDG lines. Following treatment of CDG and CDDG cell lines with optimized concentrations of candidate compounds (6, 9, 15, 26) and a non-active control compound (28), p-eIF2α and ATF6 levels were assessed by immunoblot. **A**–**C** Representative images of immunoblots. TNM, tunicamycin (50 nM). (**D**, **E**) Quantification of p-eIF2α levels in levels in CDG/CDDG treated cells. (*n* = 3 per each genotype and treatment). Expression data in (**D**, **E**) are relative to expression levels in parental RPE-1 cells. ns, not significant. *P < 0.05, **P < 0.01, ***P < 0.001, ***P < 0.001 by one-way ANOVA followed by Dunnett multiple comparisons post-test.

We then tested whether lead active compounds could alleviate markers of the other ER stress pathways dysregulated in our models (Fig. 2). We treated the CDG and CDDG lines with optimized concentrations of the top two Group I (6, 9) and Group II (15, 26) compounds as well as a non-active control compound (28), and then assessed levels of p-eIF2α and ATF6 (Fig. 7). As expected, all candidate compounds effectively decreased levels of p-eIF2α and decreased ATF6(90) cleavage relative to vehicle-treated control *NGLY1*^−/−^ cells (Fig. 7A, D, E). The *PMM2*^F119L/^^−^ line did not exhibit abnormal levels of p-eIF2α or ATF6(90) and none of the candidate compounds adversely affected expression of these markers (Fig. 7B, D, E). Similar to *NGLY1*^−/−^, all candidate compounds were able to effectively alleviate markers of ER stress in the *DPAGT1*^+/−^ line (Fig. 7C–E). We note that the non-active control compound tested (#28) was able to reduce p-eIF2α levels in *DPAGT1*^+/−^ cells; however, it was not as effective as candidate compounds (Fig. 7E). We also note that, despite an indication of autophagy induction by Group II compounds 15 and 26 in the primary screen, we did not see a significant increase (or reduction) of SQSTM1/p62 protein levels following treatment with any of the tested compounds in any of the cellular models (Supplementary Fig. 4).

Table 1 provides a summary of correction for proliferation and ER stress markers for each compound tested in each of the CDG and CDDG lines. Taken together, our screen identified several highly-effective lead compounds that are able to correct proliferation defects as well as alleviate molecular markers of multiple ER stress pathways dysregulated in cellular models of CDG and CDDG.

**Table 1:** Correlation between the proliferation, sXBP1 and ATF6 expression and peIF2α levels in CDG and CDDG cell lines treated with candidate and non-active control compounds.

Compounds	*NGLY1*^−^^*/−*^				*PMM2*^*F119L/−*^				*DPAGT1*^*+/−*^			
	Proliferation	*sXBP1*	peIF2α	ATF6	Proliferation	*sXBP1*	peIF2α^a^	ATF6^a^	Proliferation	*sXBP1*	peIF2α	ATF6
Candidates Group I												
1	**	ns			ns	ns			ns	ns		
2	****	ns			ns	ns			ns	ns		
3	ns	****			***	ns			****	*		
4	ns	****			ns	ns			ns	**		
5	ns	*			*	ns			ns	ns		
6	****	****	****	***	****	**	ns	ns	***	**	****	****
9	***	****	****	*	***	**	ns	ns	****	***	****	****
Candidates Group II												
14	ns	****			ns	ns			ns	***		
15	****	****	****	**	****	**	ns	ns	****	****	****	****
16	***	ns			ns	ns			ns	ns		
18	****	ns			ns	ns			ns	ns		
25	ns	****			****	**			****	****		
26	ns	****	****	**	****	***	ns	ns	****	****	****	***
Non-active controls												
28	ns	ns	ns	ns	ns	ns	ns	ns	ns	ns	****	ns
29	ns	ns			ns	ns			ns	ns		
30	ns	ns			ns	ns			ns	ns		
31	ns	ns			ns	ns			ns	ns		
32	ns	ns			ns	ns			ns	ns		
33	ns	ns			ns	ns			ns	ns		
34	ns	ns			ns	ns			ns	ns		
35	ns	ns			ns	ns			ns	ns		
36	ns	ns			ns	ns			ns	ns		
37	ns	ns			ns	ns			ns	ns		
38	ns	ns			ns	ns			ns	ns		

^a^No phenotype for these ER stress markers.

*P < 0.05, **P < 0.01, ***P < 0.001, ****P < 0.0001.

## Discussion

A central challenge to the development of novel therapies is the availability of screenable models that focus on disease-relevant phenotypes. Screens based on mutation-induced phenotypes, such as morphological differences, allows one to establish a screening assay without a full understanding of the molecular mechanisms that drive disease pathology. This creates an opportunity for the identification of new therapeutic targets as well as uncovering new insights related to etiology.

The objective of the high-content, phenotypic screen described here was to rapidly identify small molecules capable of alleviating ER stress in cellular models of monogenic disease. The rationale for our screen is that ER stress responses should be applicable across a variety of cell types, and drugs capable of alleviating ER stress will help treat symptoms of disease. Backed by the growing body of evidence linking ER stress to multiple neurological conditions and to CDG and CDDG, we reasoned that using ER stress markers as a functional readout combined with cellular phenotypes can serve as a proxy for overall cellular health on a disease background. It was important to develop the CDDG and CDG models in a cell type with uniform morphology that permits rapid and easily quantifiable morphology changes. We note that a screen in a more disease-relevant cell type such as hiPSC-derived neurons may be more applicable; however, such approaches have a number of drawbacks that our approach addresses. For instance, common neuronal differentiation methods yield a heterogeneous population of cells with differing levels of maturity and morphology that renders potential molecular or morphological phenotypes difficult to identify or interpret. Moreover, the labor intensiveness and cost of differentiation methods often makes large-scale screens prohibitive. Rather, a multi-tiered strategy whereby large screens are performed on genetic cellular models with high confidence phenotypes and lead compounds are then validated in more relevant cellular and/or animal models is more efficacious.

A majority of the active compounds in our screen, #s 3, 4, 6, 9, 15, 25, are reported to affect microtubules (Supplementary Table 3) either through direct effects on microtubules themselves or by targeting proteins (e.g. kinases) that regulate microtubule dynamics [30–36]. Compounds structurally similar to compounds 6, 9, 15, 25 and 26 (Supplementary Table 4) identified here are reported to prevent ER stress in multiple cellular systems through modulation of JAK/STAT and growth factor signaling among others (Supplementary Table 3) [37–41]. Multiple compounds converging on a biological process (the regulation of microtubules) suggests this is a legitimate therapeutic avenue.

Our study describes, to our knowledge, the first example of a high-throughput screen on genetically modified human cells for three monogenic diseases with a shared endogenous molecular phenotype. Here we focused on a biological process, ER stress, thought to unite a number of rare and more common diseases, and successfully identified bioactive ER stress diminishing compounds through unbiased morphological screening. This work has shown it is possible to develop cellular models that possess screenable phenotypes able to identify compounds that alleviate molecular and morphological phenotypes caused by the underlying genetic mutation, thereby establishing a platform to identify targeted and common treatments for monogenic disorders. Due to the genetic heterogeneity of CDGs, it will be important to determine whether the compounds identified here also alleviate ER stress-related phenotypes in other genetic causes of CDG.

Beyond establishing a paradigm for identifying therapeutic compounds for rare monogenic diseases, this work suggests a direction for identifying compounds able to alleviate the symptoms related to ER stress in more common diseases characterized by ER stress including neurodegenerative diseases. Loss of microtubule mass or altered microtubule dynamics in axons and dendrites are major contributors to neurodegenerative diseases such as ALS, Parkinson’s disease, Alzheimer’s disease, and several tauopathies [42]. Future studies will determine whether compounds that affect microtubule dynamics are able to prevent disease-relevant phenotypes in cellular models of neurodegenerative diseases.

## Supplementary information

Supplemental Material
