# Supplementary material for: Precision genetic cellular models identify therapies protective against ER stress
Source: Cell Death Dis. 2021 Aug 5;12(8):770. doi: 10.1038/s41419-021-04045-4 (PMC8342410; doi:10.1038/s41419-021-04045-4)
Supplement: Supplementary file 1 — Supplemental Material [file 41419_2021_4045_MOESM1_ESM.docx]

**List of Supplementary Materials**

Four Supplementary Figures:

**Supplementary Figure 1.**  Characterization of additional CDG and CDDG genome-edited lines.

**Supplementary Figure 2.**  CDG and CDDG lines exhibit elevated autophagy levels.

**Supplementary Figure 3.** Reversion of cellular morphology by candidate compounds.

**Supplementary Figure 4.** Select active compounds do not affect autophagy in CDG or CDDG models.

Four Supplementary Tables**:**

**Supplementary Table 1.** Primary and secondary antibody used in the study

**Supplementary Table 2.** A list of “hit” compounds determined by HTS cell-painting assay and nominated for further biochemical testing

**Supplementary Table 3.** Chemical names and properties of compounds with confirmed biological activity.

**Supplementary Table 4.** Predicted structures for compounds with confirmed biological activity.

**Supplementary References**

**Supplementary Figure 1**


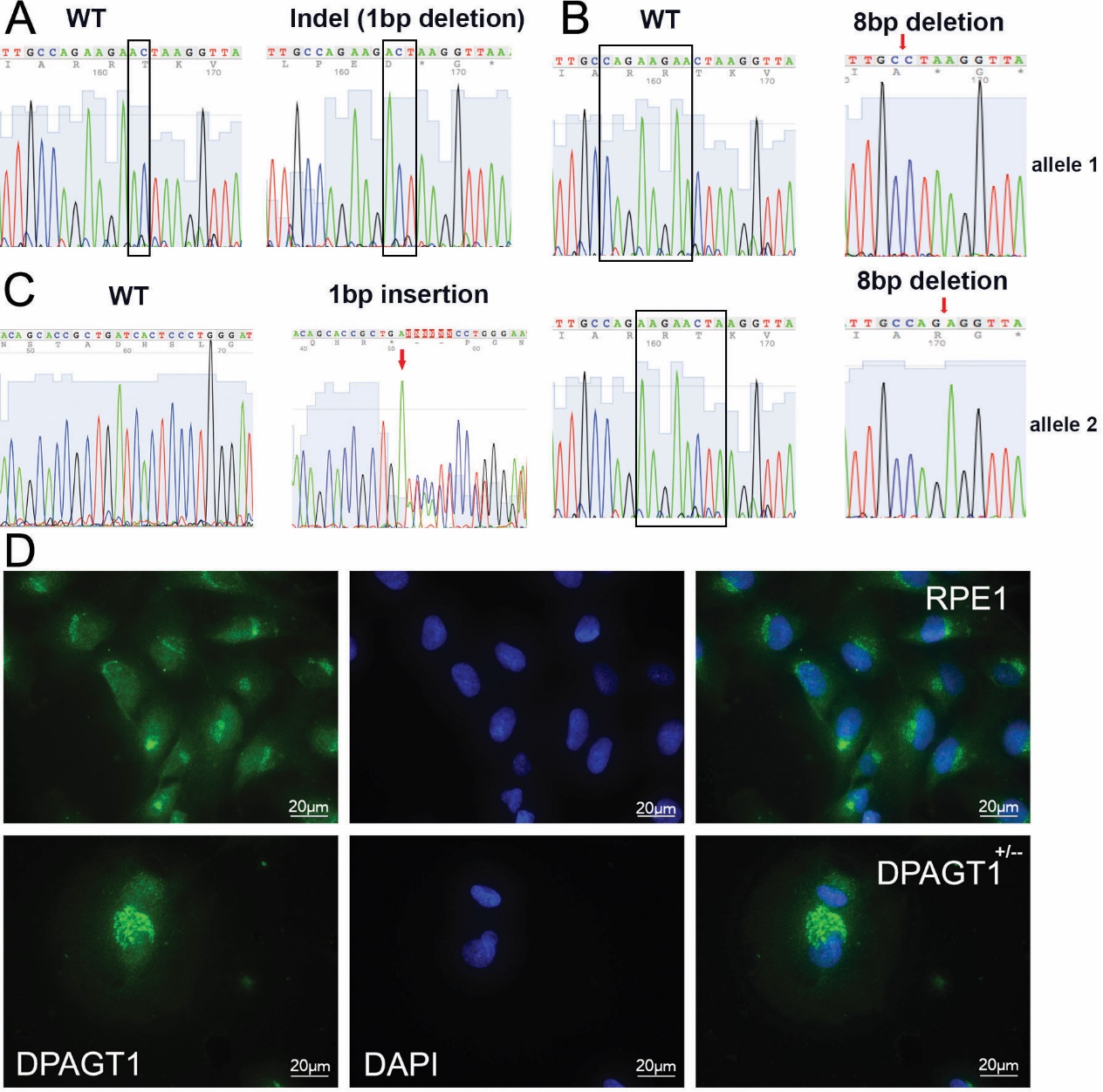

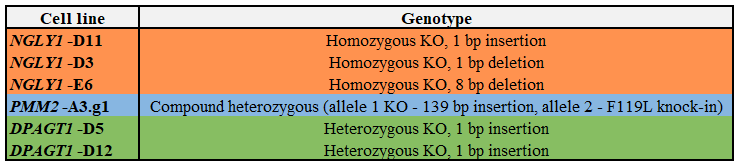


E

**Supplementary Figure 1.**  Characterization of additional CDG and CDDG genome-edited lines**.**

A-C, Electropherogram traces for parental RPE-1 cells and CDDG, *NGLY1*^-/-^ D3 (A) and *NGLY1*^-/-^ E6 (B), and CDG *DPAGT1*^+/-^ D12 (C) lines. D, Representative immunofluorescence images for staining for DPAGT1 protein in parental RPE-1 and CDG *DPAGT1*^+/-^ D12 lines. Scale bars: 20 µm. (E) Summary of genome edited CDG and CDDG cell line genotypes. All genotypes confirmed by Sanger sequencing.

**
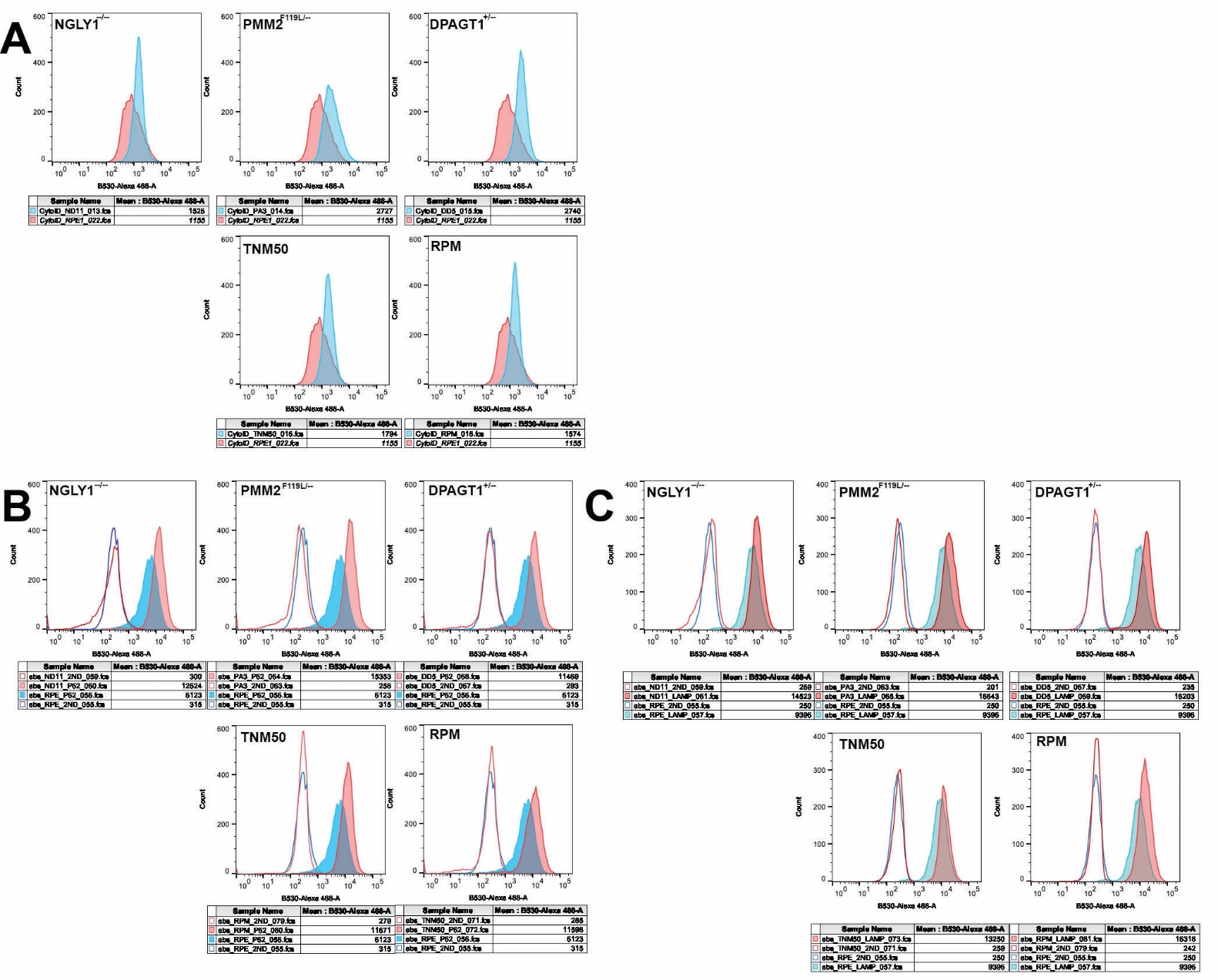
Supplementary Figure 2**

**Supplementary Figure 2.** CDG and CDDG lines exhibit elevated autophagy. **.** Representative histogram overlays of RPE-1 and isogenic CDG and CDDG cell lines stained with Cyto-ID® reagent (A), anti-p62/SQATM1 (B) and anti-LAMP1 (C) antibodies. Median Fluorescence Intensity (MFI) values for each sample are indicated in the tables below the images.

**Supplementary Figure 3**

**
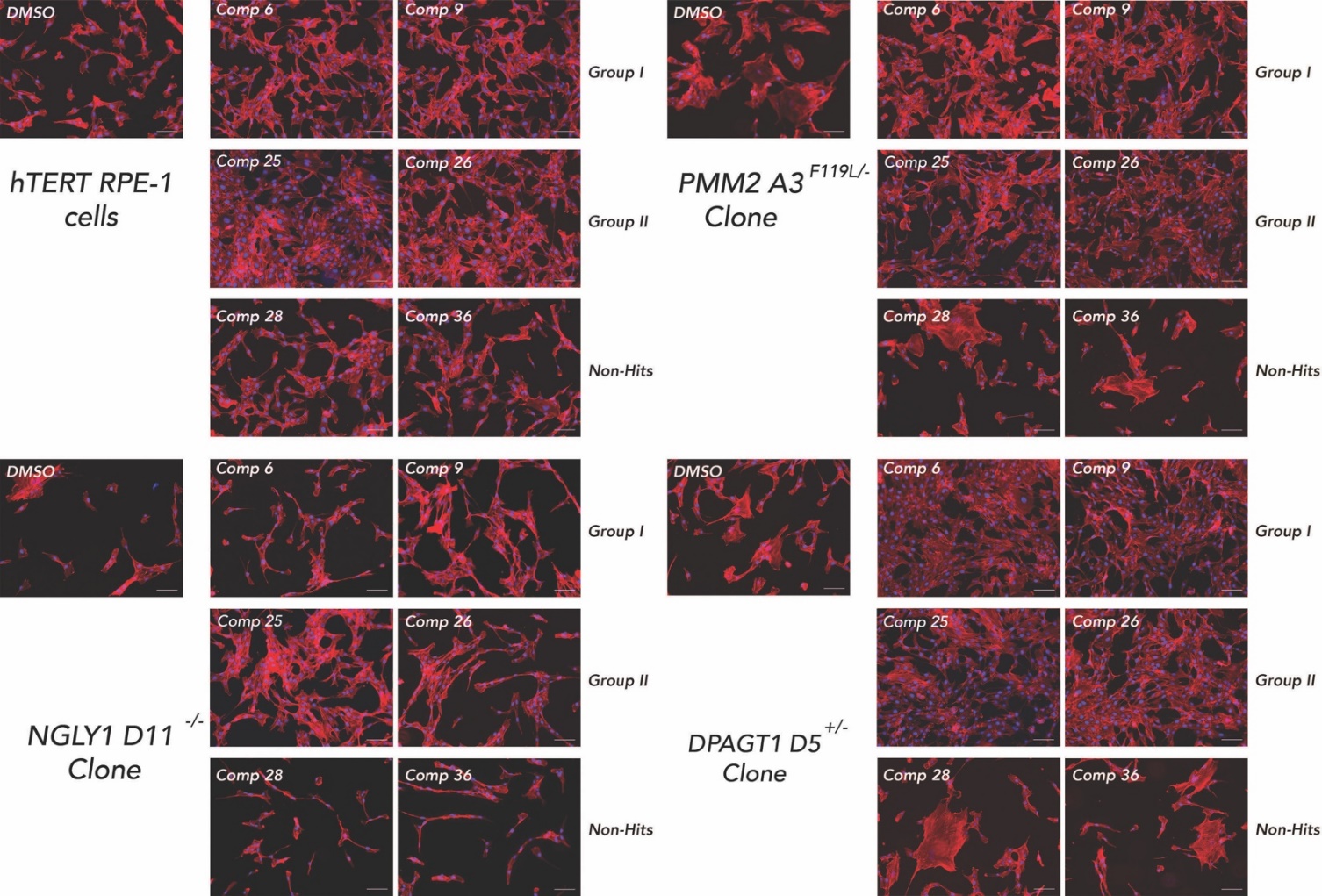
**

**Supplementary Figure 3.** **Reversion of cellular morphology by candidate compounds.** Representative images of morphological changes in parental RPE-1 and isogenic CDDG, *NGLY1*^-/-^ D11, and CDG *PMM2* ^F119L/-^ A3 and *DPAGT1*^+/-^ D5 lines upon treatment with indicated compounds.

**
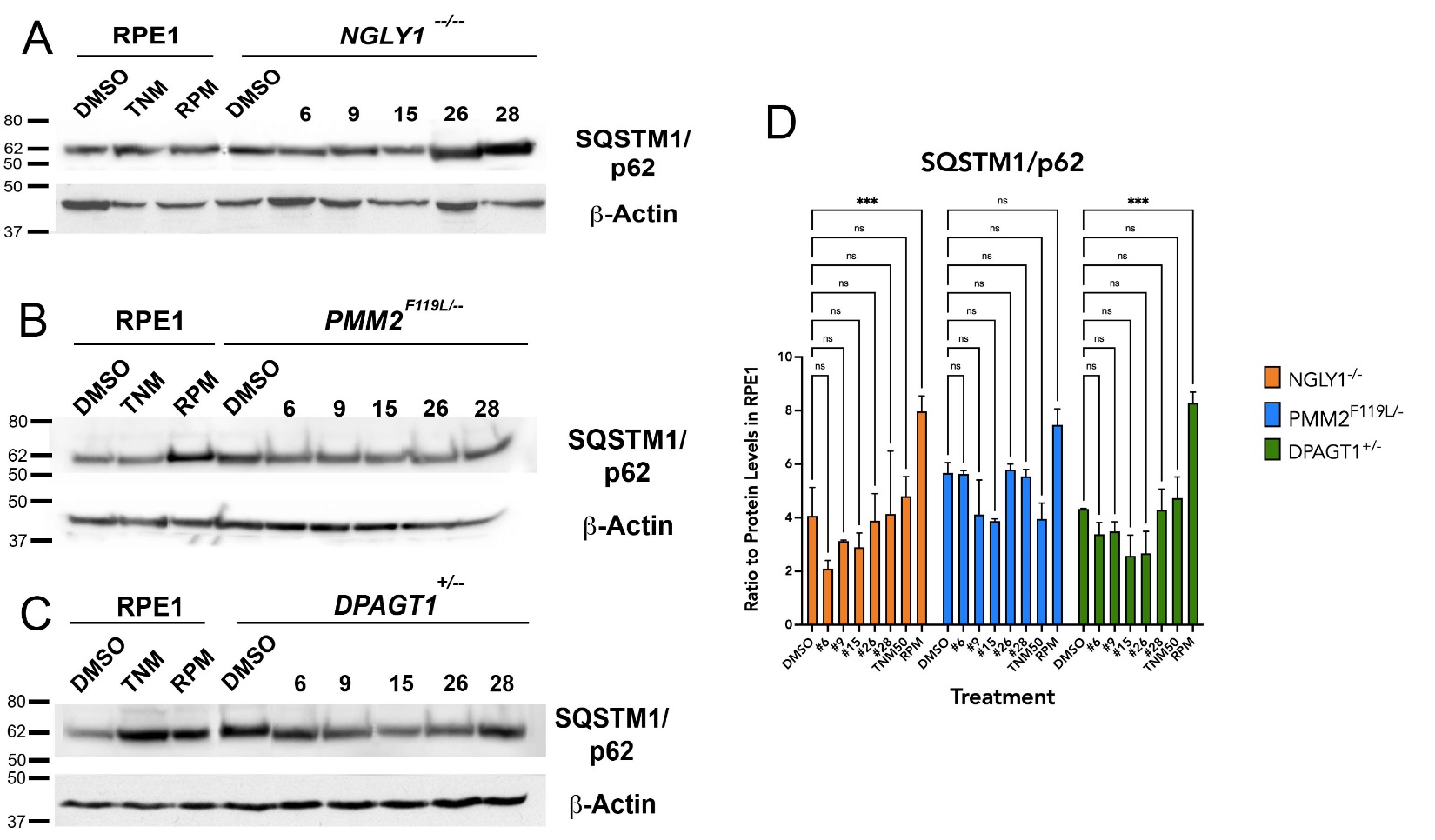
Supplementary Figure 4. ­**

**Supplementary Figure 4. Select active compounds do not affect autophagy in CDG or CDDG models.** A-C, Representative immunoblots of SQSTM1/p62 in RPE1 cells treated with either DMSO, tunicamycin (TNM, 50 nM) to induce ER stress, or rapamycin (RPM, 500 nM) to induce autophagy, and *NGLY1*^-/-^, *PMM2*^F119/-^, *DPAGT1*^+/-^ lines treated with DMSO, or optimized concentrations of Group I compounds (6, 9), Group II compounds (15, 26) or non-active control compound 28. D, Quantification of SQSTM1/p62 levels in CDG / CDDG treated cells. (n=3 per each genotype and treatment). Expression data in (D) are relative to expression in parental RPE-1 cells. ns, not significant. ***, P<0.001 by one-way ANOVA followed by Dunnett multiple comparisons post-test.

**Supplementary Table 1. Primary and secondary antibodies used in the study**

| Antibody Type | Antibody Name/Clone | Source | Manufacturer$ Cat.# | Dilutions | | |
| --- | --- | --- | --- | --- | --- | --- |
|  |  |  |  | WB | IF | FCM |
| Primary | p-eIF2a (Ser 51) | Rabbit polyclonal | Cell Signaling Technology, cat.9721 | 1:1000 | NA | MA |
|  | eIF2a | Rabbit polyclonal | Cell Signaling Technology, cat.9722 | 1:1000 | NA | NA |
|  | NGLY1 | Rabbit polyclonal | Bethyl Laboratories, cat.A305-547A-T | 1:1000 | NA | NA |
|  | ATF6 (70B1413.1) | Mouse monoclonal | Novus Biologicals, cat.NBP1-40256 | 1:500 | NA | NA |
|  | PMM2 (2E9) | Mouse monoclonal | Novus Biologicals, cat.H00005373-M01 | 1:200 | 1:500 | NA |
|  | DPAGT1 | Rabbit polyclonal | Abcam, cat.ab116667 | 1:100 | 1:500 | NA |
|  | CHOP (L637F) | Mouse monoclonal | Cell Signaling Technology, cat.2895 | NA | 1:3200 | NA |
|  | GADD153/CHOP | Rabbit polyclonal | Novus Biologicals, cat.NBP2-58505 | NA | 1:500 | NA |
|  | LAMP1/CD17a (H4A3) | Mouse monoclonal | Novus Biologicals, cat.NBP2-25183 | NA | 1:500 | 1:500 |
|  | SQSTM1/p62 | Mouse monoclonal | Abcam, cat.ab56416 | NA | 1:200 | 1:1000 |
|  | P4HB/PDIA1 | Rabbit polyclonal | Abcam, cat.ab3672 | NA | 1:200 | NA |
|  | HRP-b-Actin | Mouse monoclonal | Santa Cruz Biotechnology, cat.sc-47778HRP | 1:10000 | NA | NA |
|  | IgG1 Isotype Control (NCG01) | Mouse monoclonal | ThermoFisher Scientific, cat. A32723 | NA | NA | 1:500 |
| Secondary | IgG (H+L) Poly-HRP | Goat anti-Rabbit | ThermoFisher Scientific, cat. 32260 | 1:10000 | NA | NA |
|  | IgG (H+L) Poly-HRP | Goat anti-Mouse | ThermoFisher Scientific, cat. 31430 | 1:10000 | NA | NA |
|  | IgG (H+L) Alexa Fluor Plus 488 | Donkey anti-Mouse | Invitrogen, cat.A32766 | NA | 1:1000 | NA |
|  | IgG (H+L) Alexa Fluor Plus 488 | Donkey anti-Rabbit | Invitrogen, cat.A32790 | NA | 1:1000 | NA |
|  | IgG (H+L) Alexa Fluor Plus 568 | Donkey anti-Rabbit | Invitrogen, cat.A10042 | NA | 1:1000 | NA |
|  | IgG (H+L) Alexa Fluor Plus 568 | Donkey anti-Mouse | Invitrogen, cat.A10037 | NA | 1:1000 | NA |
|  | IgG (H+L) Alexa Fluor Plus 488 | Goat anti-Mouse | Invitrogen, cat.A32723 |  |  | 1:1000 |

**Supplementary Table 2.**

A list of “hit” compounds determined by HTS cell-painting assay and nominated for further biochemical testing

|  | Primary Screen Hit (n=1) | | | Confirmation (n=4) | | | LC3 puncta (% control) | | |
| --- | --- | --- | --- | --- | --- | --- | --- | --- | --- |
| Index | *DPAGT1* | *NGLY1* | *PMM2* | *DPAGT1* | *NGLY1* | *PMM2* | *DPAGT1* | *NGLY1* | *PMM2* |
| 1 | 1 | 1 | 1 | 4 | 4 | 1 | 31 | 30 | 23 |
| 2 |  | 1 | 1 | 4 | 2 |  | 22 | 11 | 8 |
| 3 | 1 | 1 | 1 | 3 | 4 |  | 27 | 13 | 11 |
| 4 | 1 | 1 | 1 | 3 | 3 |  | 27 | 18 | 18 |
| 5 |  | 1 | 1 | 2 | 2 | 1 | 27 | 14 | 15 |
| 6 |  | 1 | 1 | 2 | 2 |  | 29 | 27 | 30 |
| 7 | 1 | 1 |  | 2 | 1 |  | 42 | 30 | 19 |
| 8 | 1 | 1 |  | 1 | 4 |  | 47 | 10 | 13 |
| 9 |  | 1 | 1 | 1 | 2 | 1 | 27 | 16 | 11 |
| 10 | 1 | 1 | 1 |  |  |  | 26 | 13 | 12 |
| 11 | 1 | 1 | 1 |  |  |  | 37 | 18 | 10 |
| 12 |  | 1 | 1 |  |  |  | 39 | 32 | 30 |
|  |  |  |  |  |  |  |  |  |  |
| 14 | 1 | 1 |  | 4 | 3 |  | 95 | 47 | 35 |
| 15 | 1 | 1 |  | 4 | 2 |  | 85 | 55 | 29 |
| 16 | 1 | 1 | 1 | 3 | 4 |  | 80 | 34 | 40 |
| 18 | 1 | 1 |  | 3 | 3 |  | 131 | 63 | 51 |
| 19 |  | 1 | 1 | 3 | 2 |  | 105 | 66 | 64 |
| 21 | 1 | 1 |  | 2 | 3 |  | 85 | 40 | 33 |
| 24 | 1 | 1 | 1 | 1 | 3 |  | 109 | 37 | 34 |
| 25 |  | 1 | 1 | 1 | 3 | 1 | 117 | 48 | 52 |
| 26 | 1 | 1 |  | 1 | 2 | 1 | 96 | 45 | 37 |
| 27 |  | 1 | 1 |  | 2 |  | 108 | 42 | 26 |

**Supplementary Table 3.**

Chemical names and properties of compounds with confirmed biological activity.

| Index | CAS Number | IUPAC Name | Name* and/or Chemical Class | Target |
| --- | --- | --- | --- | --- |
| 3 | 31430-18-9 | methyl N-[5-(thiophene-2-carbonyl)-1H-benzimidazol-2-yl]carbamate | Nocodazole  (Benzimidazoles) | Microtubules ^1, 2, 3^ |
| 4 | 14255-87-9 | methyl N-(5-butyl-1H-benzimidazol-2-yl) carbamate | Parbendazole (Benzimidazoles) | Microtubules ^4, 5, 6^ |
| 6 | 840534-89-6 | 2-[3-[[3-(3-fluorophenyl)triazolo[4,5-d]pyrimidin-5-yl] amino]phenyl]acetic acid | Triazolopyrimidines | Microtubules ^7^, NOX2 ^8^, TDP2 ^9^, GSN2, PERK, HRI, IRE1 inhibitor ^10, 11^ |
| 9 | 518-28-5 | (5R,5aR,8aR,9R)-5-hydroxy-9-(3,4,5-trimethoxyphenyl)-5a,6,8a,9-tetrahydro-5H-isobenzofuro[6,5-f][1,3]benzodioxol-8-one | Podophyllotoxin (Lignans,  Furonaphthodioxols) | Microtubules ^12, 13^, IGF 1R ^14, 15^, IDH1 ^16^, TDP1/2 ^17^ |
| 15 | 935666-88-9 | 5-chloro-N2-[(1S)-1-(5-fluoropyrimidin-2-yl)ethyl]-N4-(5-methyl-1H-pyrazol-3-yl)pyrimidine-2,4-diamine | AZD1480  (Aminopyrazoles) | Microtubules ^18^, JAK1/2 ^18, 19, 20^, ALK, LTK, FGFR, RET and TRK kinases inhibitor ^21^, STAT3 and STAT5A inhibitor ^22, 23^, IDH1 inhibitor ^19^ |
| 25 | 627517-32-2 | [3-(3-fluoroanilino)-6,7-dimethoxy-4H-indeno[1,2-c]pyrazol-1-yl]methyl butanoate | Aminopyrazoles  Benzenesulfonamides | Microtubules ^24, 25, 26, 27^  PDGF-R kinase inhibitor ^28^ |
| 26 | 842128-50-1 | 3-[5-[3-(2-aminopyrimidin-4-yl)anilino]triazolo[4,5-d]pyrimidin-3-yl]benzenesulfonamide | Aminopyrimidines, benzensulfonamides | Possible CDK1/2/5/9 inhibitor ^29, 30, 31, 32^ |

*Commercial name if available

**Supplementary Table 4.**

Predicted structures for compounds with confirmed biological activity. Group I compounds (left), Group II compounds (right).

| **Index** | **CAS No.** | **GCRS Sketch** | **SMILES** |
| --- | --- | --- | --- |
| 1 | 1092504-43-2 | 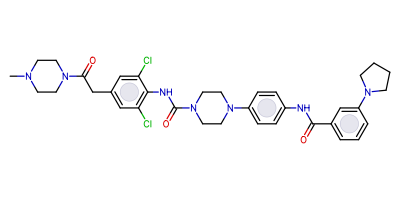 | 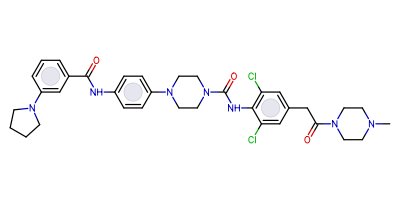 |
| 2 | 126452-70-8 | 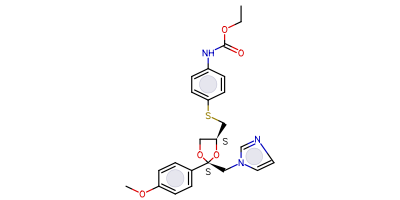 | 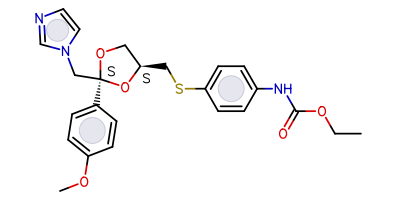 |
| 3 | 31430-18-9 | 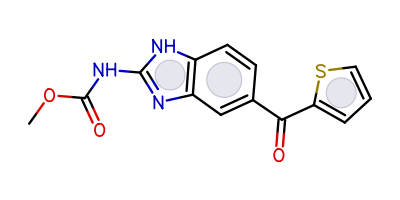 | 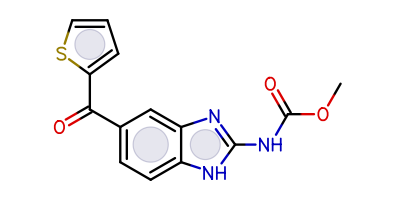 |
| 4 | 14255-87-9 | 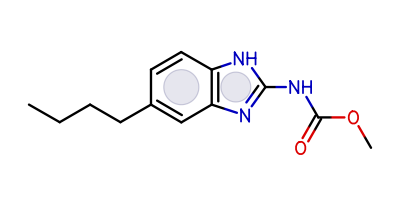 | 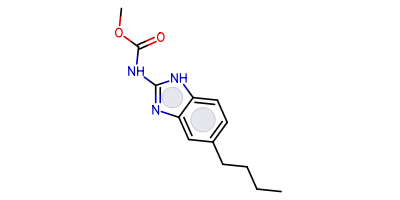 |
| 5 | 627512-62-3 | 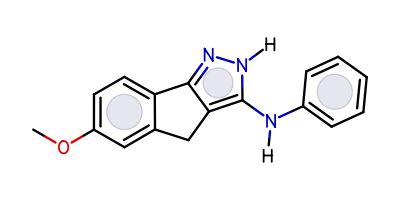 | 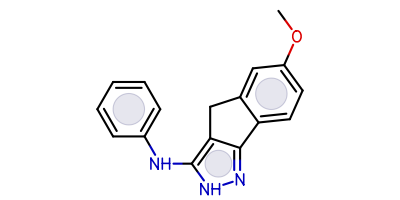 |
| 6 | 840534-89-6 | 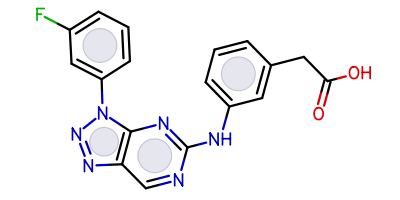 | 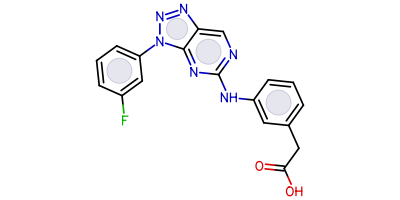 |
| 9 | 518-28-5 | 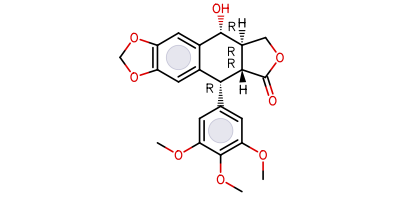 | 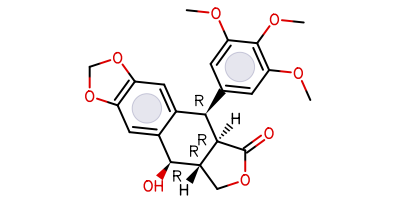 |

| **Index** | **CAS No.** | **GCRS Sketch** | **SMILES** |
| --- | --- | --- | --- |
| 14 | 443798-87-6 | 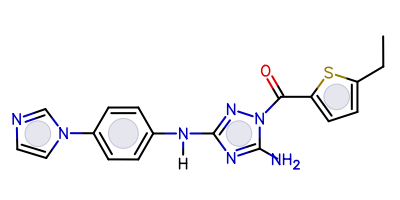 | 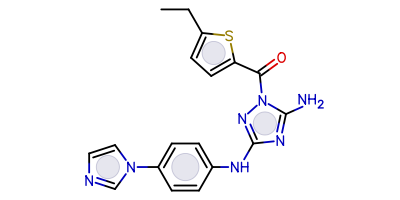 |
| 15 | 935666-88-9 | 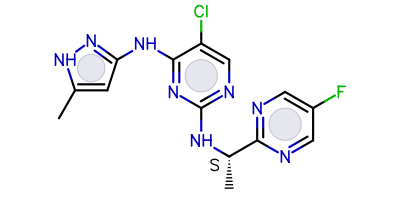 | 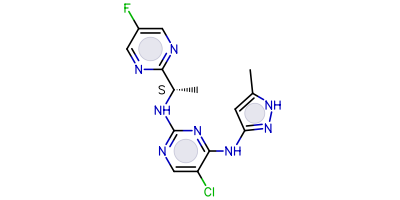 |
| 16 | 244768-00-1 | 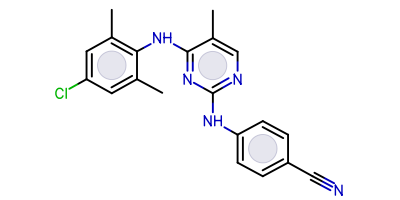 | 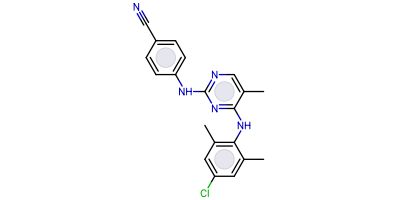 |
| 18 | 443799-16-4 | 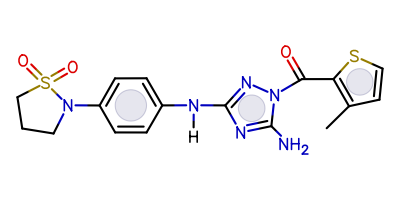 | 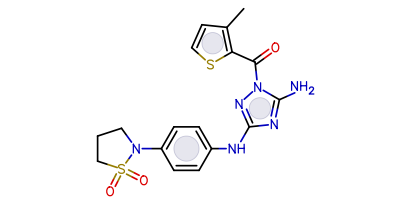 |
| 25 | 244767-84-8 | 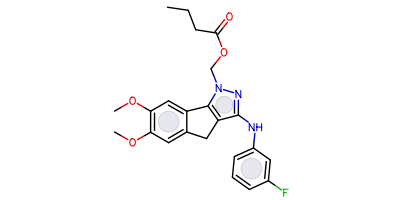 | 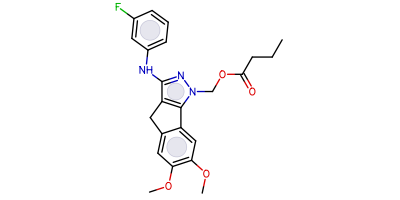 |
| 26 | 627517-32-2 | 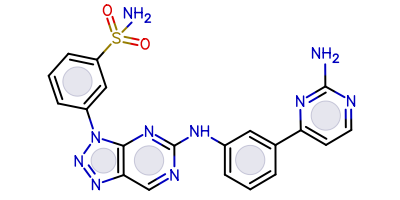 | 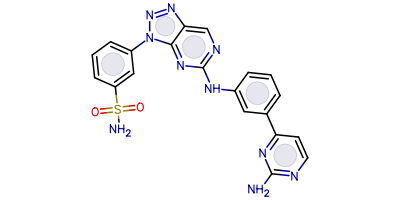 |

**Supplementary References.**

1. Kadir S, Astin JW, Tahtamouni L, Martin P, Nobes CD. Microtubule remodelling is required for the front-rear polarity switch during contact inhibition of locomotion. *J Cell Sci* 2011, **124**(Pt 15)**:** 2642-2653.

2. Rochlin MW, Wickline KM, Bridgman PC. Microtubule stability decreases axon elongation but not axoplasm production. *J Neurosci* 1996, **16**(10)**:** 3236-3246.

3. Sengottuvel V, Fischer D. Facilitating axon regeneration in the injured CNS by microtubules stabilization. *Commun Integr Biol* 2011, **4**(4)**:** 391-393.

4. Jordan MA. Mechanism of action of antitumor drugs that interact with microtubules and tubulin. *Curr Med Chem Anticancer Agents* 2002, **2**(1)**:** 1-17.

5. Brum AM, van de Peppel J, van der Leije CS, Schreuders-Koedam M, Eijken M, van der Eerden BC*, et al.* Connectivity Map-based discovery of parbendazole reveals targetable human osteogenic pathway. *Proc Natl Acad Sci U S A* 2015, **112**(41)**:** 12711-12716.

6. Ganguly A, Zhang H, Sharma R, Parsons S, Patel KD. Isolation of human umbilical vein endothelial cells and their use in the study of neutrophil transmigration under flow conditions. *J Vis Exp* 2012(66)**:** e4032.

7. Oukoloff K, Kovalevich J, Cornec AS, Yao Y, Owyang ZA, James M*, et al.* Design, synthesis and evaluation of photoactivatable derivatives of microtubule (MT)-active [1,2,4]triazolo[1,5-a]pyrimidines. *Bioorg Med Chem Lett* 2018, **28**(12)**:** 2180-2183.

8. Sun QA, Hess DT, Wang B, Miyagi M, Stamler JS. Off-target thiol alkylation by the NADPH oxidase inhibitor 3-benzyl-7-(2-benzoxazolyl)thio-1,2,3-triazolo[4,5-d]pyrimidine (VAS2870). *Free Radic Biol Med* 2012, **52**(9)**:** 1897-1902.

9. Ribeiro CJA, Kankanala J, Xie J, Williams J, Aihara H, Wang Z. Triazolopyrimidine and triazolopyridine scaffolds as TDP2 inhibitors. *Bioorg Med Chem Lett* 2019, **29**(2)**:** 257-261.

10. Lough L, Sherman D, Beccera-Flores M, Lavinda O, Ni E, Wang H*, et al.* Triazolo[4,5-d]pyrimidines as Validated General Control Nonderepressible 2 (GCN2) Protein Kinase Inhibitors Reduce Growth of Leukemia Cells. *Comput Struct Biotechnol J* 2018, **16:** 350-360.

11. Dorsch D, Hoelzemann G, Schiemann K, Wegener A, inventors; Merck Patent GmbH, assignee. Triazolo[4,5-d]pyrimidine derivatives. United States patent 9,409,914. 2016.

12. Jordan MA, Thrower D, Wilson L. Effects of vinblastine, podophyllotoxin and nocodazole on mitotic spindles. Implications for the role of microtubule dynamics in mitosis. *J Cell Sci* 1992, **102 ( Pt 3):** 401-416.

13. Sahenk Z, Brady ST. Axonal tubulin and microtubules: morphologic evidence for stable regions on axonal microtubules. *Cell Motil Cytoskeleton* 1987, **8**(2)**:** 155-164.

14. Menu E, Jernberg-Wiklund H, De Raeve H, De Leenheer E, Coulton L, Gallagher O*, et al.* Targeting the IGF-1R using picropodophyllin in the therapeutical 5T2MM mouse model of multiple myeloma: beneficial effects on tumor growth, angiogenesis, bone disease and survival. *Int J Cancer* 2007, **121**(8)**:** 1857-1861.

15. Rosengren L, Vasilcanu D, Vasilcanu R, Fickenscher S, Sehat B, Natalishvili N*, et al.* IGF-1R tyrosine kinase expression and dependency in clones of IGF-1R knockout cells (R-). *Biochem Biophys Res Commun* 2006, **347**(4)**:** 1059-1066.

16. Bala Bhaskara Rao K, Katragunta K, Sarma UM, Jain N. Abundance of d-2-hydroxyglutarate in G2/M is determined by FOXM1 in mutant IDH1-expressing cells. *FEBS Lett* 2019, **593**(16)**:** 2177-2193.

17. Kankanala J, Ribeiro CJA, Kiselev E, Ravji A, Williams J, Xie J*, et al.* Novel Deazaflavin Analogues Potently Inhibited Tyrosyl DNA Phosphodiesterase 2 (TDP2) and Strongly Sensitized Cancer Cells toward Treatment with Topoisomerase II (TOP2) Poison Etoposide. *J Med Chem* 2019, **62**(9)**:** 4669-4682.

18. Gu L, Talati P, Vogiatzi P, Romero-Weaver AL, Abdulghani J, Liao Z*, et al.* Pharmacologic suppression of JAK1/2 by JAK1/2 inhibitor AZD1480 potently inhibits IL-6-induced experimental prostate cancer metastases formation. *Mol Cancer Ther* 2014, **13**(5)**:** 1246-1258.

19. Ioannidis S, Lamb ML, Wang T, Almeida L, Block MH, Davies AM*, et al.* Discovery of 5-chloro-N2-[(1S)-1-(5-fluoropyrimidin-2-yl)ethyl]-N4-(5-methyl-1H-pyrazol-3-yl)p yrimidine-2,4-diamine (AZD1480) as a novel inhibitor of the Jak/Stat pathway. *J Med Chem* 2011, **54**(1)**:** 262-276.

20. Qin H, Buckley JA, Li X, Liu Y, Fox TH, 3rd, Meares GP*, et al.* Inhibition of the JAK/STAT Pathway Protects Against alpha-Synuclein-Induced Neuroinflammation and Dopaminergic Neurodegeneration. *J Neurosci* 2016, **36**(18)**:** 5144-5159.

21. Gudernova I, Balek L, Varecha M, Kucerova JF, Kunova Bosakova M, Fafilek B*, et al.* Inhibitor repurposing reveals ALK, LTK, FGFR, RET and TRK kinases as the targets of AZD1480. *Oncotarget* 2017, **8**(65)**:** 109319-109331.

22. Hedvat M, Huszar D, Herrmann A, Gozgit JM, Schroeder A, Sheehy A*, et al.* The JAK2 inhibitor AZD1480 potently blocks Stat3 signaling and oncogenesis in solid tumors. *Cancer Cell* 2009, **16**(6)**:** 487-497.

23. Meares GP, Liu Y, Rajbhandari R, Qin H, Nozell SE, Mobley JA*, et al.* PERK-dependent activation of JAK1 and STAT3 contributes to endoplasmic reticulum stress-induced inflammation. *Mol Cell Biol* 2014, **34**(20)**:** 3911-3925.

24. Chen J, Sun WL, Wasylyk B, Wang YP, Zheng H. c-Jun N-terminal kinase mediates microtubule-depolymerizing agent-induced microtubule depolymerization and G2/M arrest in MCF-7 breast cancer cells. *Anticancer Drugs* 2012, **23**(1)**:** 98-107.

25. Minegishi H, Futamura Y, Fukashiro S, Muroi M, Kawatani M, Osada H*, et al.* Methyl 3-((6-methoxy-1,4-dihydroindeno[1,2-c]pyrazol-3-yl)amino)benzoate (GN39482) as a tubulin polymerization inhibitor identified by MorphoBase and ChemProteoBase profiling methods. *J Med Chem* 2015, **58**(10)**:** 4230-4241.

26. Tsyganov DV, Khrustalev VN, Konyushkin LD, Raihstat MM, Firgang SI, Semenov RV*, et al.* 3-(5-)-Amino-o-diarylisoxazoles: regioselective synthesis and antitubulin activity. *Eur J Med Chem* 2014, **73:** 112-125.

27. Tsyganov DV, Konyushkin LD, Karmanova IB, Firgang SI, Strelenko YA, Semenova MN*, et al.* cis-Restricted 3-aminopyrazole analogues of combretastatins: synthesis from plant polyalkoxybenzenes and biological evaluation in the cytotoxicity and phenotypic sea urchin embryo assays. *J Nat Prod* 2013, **76**(8)**:** 1485-1491.

28. Ho CY, Brunmark BA, Emanuel S, Galemmo J, Robert A, Johnson DL, Ludovichi DW*, et al.*, inventors; Janssen Parmaceutica N.V. (BE), assignee. N-substituted tricyclic 3-aminopyrazoles as inhibitors for the treatment of cell proliferative disorders. United States patent 7,196,110. 2007 March 27, 2007.

29. Alexander LT, Mobitz H, Drueckes P, Savitsky P, Fedorov O, Elkins JM*, et al.* Type II Inhibitors Targeting CDK2. *ACS Chem Biol* 2015, **10**(9)**:** 2116-2125.

30. Coxon CR, Anscombe E, Harnor SJ, Martin MP, Carbain B, Golding BT*, et al.* Cyclin-Dependent Kinase (CDK) Inhibitors: Structure-Activity Relationships and Insights into the CDK-2 Selectivity of 6-Substituted 2-Arylaminopurines. *J Med Chem* 2017, **60**(5)**:** 1746-1767.

31. Marchetti F, Cano C, Curtin NJ, Golding BT, Griffin RJ, Haggerty K*, et al.* Synthesis and biological evaluation of 5-substituted O4-alkylpyrimidines as CDK2 inhibitors. *Org Biomol Chem* 2010, **8**(10)**:** 2397-2407.

32. Nguyen TK, Grant S. Dinaciclib (SCH727965) inhibits the unfolded protein response through a CDK1- and 5-dependent mechanism. *Mol Cancer Ther* 2014, **13**(3)**:** 662-674.
